# Exploring the molecular interaction of mebendazole with bovine serum albumin using multi-spectroscopic approaches and molecular docking

**DOI:** 10.1038/s41598-022-15696-4

**Published:** 2022-07-08

**Authors:** Reem N. El Gammal, Heba Elmansi, Ali A. El-Emam, Fathalla Belal, Mohammed E. A. Hammouda

**Affiliations:** 1 Department of Medicinal Chemistry, Faculty of Pharmacy, Mansoura University, Mansoura, 35516 Egypt; 2 Pharmaceutical Analytical Chemistry Department, Faculty of Pharmacy, Mansoura University, Mansoura, 35516 Egypt; 3 Department of Pharmaceutical Chemistry, Faculty of Pharmacy, Horus University - Egypt (HUE), New Damietta, Egypt

**Keywords:** Chemistry, Analytical chemistry, Medicinal chemistry

## Abstract

This article presents the binding interaction between mebendazole (MBZ) and bovine serum albumin. The interaction has been studied using different techniques, such as fluorescence quenching spectroscopy, UV–visible spectroscopy, synchronous fluorescence spectroscopy, fourier transform infrared, and fluorescence resonance energy transfer in addition to molecular docking. Results from Stern Volmer equation stated that the quenching for MBZ-BSA binding was static. The fluorescence quenching spectroscopic study was performed at three temperature settings. The binding constant (k_q_), the number of binding sites (n), thermodynamic parameters (ΔH^ο^, ΔS^ο^ and ΔG^ο^), and binding forces were determined. The results exhibited that the interaction was endothermic. It was revealed that intermolecular hydrophobic forces led to the stabilization of the drug-protein system. Using the site marker technique, the binding between MBZ and BSA was found to be located at subdomain IIA (site I). This was furtherly approved using the molecular docking technique with the most stable MBZ configuration. This research may aid in understanding the pharmacokinetics and toxicity of MBZ and give fundamental data for its safe usage to avoid its toxicity.

## Introduction

Serum albumin is the main blood plasma protein that forms molecular interactions with small molecules through binding at specific sites and assisting in their transport throughout the circulation, either exogenously or endogenously^1^. Moreover, the structural similarities between bovine serum albumin (BSA) and human serum albumin (HSA) make it an essential subject in in-vitro research. BSA is composed of 583 amino acids with a molecular weight of 66.5 kDa. The protein structure of BSA is divided into three homologous subdomains that are assembled in a linear arrangement and subdivided into subdomains A and B^2–4^. BSA contains two tryptophan residues and 20 tyrosin residues. Ligands may bind to BSA at more than one site, which are situated in the hydrophobic cavities of subdomains IIA or IIIA. Because of high structural similarity with HSA, properties such as low cost, easy availability, and similarity in ligand binding patterns with HSA have led to the using of BSA as a model protein for studying the interactions between drugs and plasma proteins. The stable BSA-drug complex is the perfect model for obtaining basic insights into plasma-drug binding^5^.

The pharmacokinetics of a drug is mainly dependent on the nature of its interaction with plasma proteins, especially albumins^6,7^. It was found that the drug’s affinity to serum albumin basically controls its concentrations in the free and bound forms, however their equilibrium is critical to the drug’s mode of action. Consequently, it is crucial to reach a full understanding of the drug’s binding mechanism with serum albumin to avoid its toxic effects^8^.

**Figure 1:**
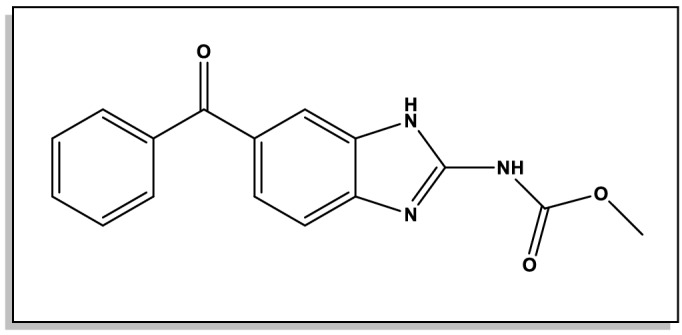
Structural formula of Mebendazole (MBZ).

Mebendazole (MBZ) is a benzimidazole carbamate derivative (Fig. 1). It is highly lipophilic (log P 2.83) with low solubility. It undergoes extensive first-pass metabolism^9^. MBZ is poorly absorbed from the gastrointestinal tract; it is used orally to treat intestinal nematode infections such as ascariasis, enterobiasis, hookworm, and trichuriasis^9^. It inhibits or destroys cytoplasmic microtubules in the worm’s intestinal or absorptive cells. Besides, it acts through inhibition of glucose uptake and depletion of glycogen stores leading to worm’s death within several days^9^. Mebendazole is a novel cancer medicine focusing on cells that are resistant to existing treatments. It exhibits cytotoxic activity, which synergizes with ionizing radiations and different chemotherapeutic agents and stimulates an antitumoral immune response^10^.

In the case of mebendazole overdose, it exerts acute oral toxicity, and its LD_50_ is 620 mg/kg. Symptoms of overdose include elevated liver enzymes, headaches, hair loss, low levels of white blood cells (neutropenia), fever, and itching^11^. It is evident that it seems very important to study the drug’s binding mechanism with serum albumin to avoid its toxic effects.

In this in-vitro study, the interaction between MBZ and BSA has been studied using the quenching fluorescence method. Thermodynamic parameters were computed to obtain binding constants at various temperature settings using Tris–HCl (pH 7.4). As well, the binding forces and the binding sites were determined. UV–visible spectroscopy, synchronous fluorescence (SF), and FTIR were used to investigate changes in protein structure. The interaction was indicated to be endothermic. Using the site marker technique, the binding between MBZ and BSA was found to be located at subdomain IIA (site I). This was also confirmed using molecular docking technique with the most stable MBZ configuration. So, these results proved the binding relationship between MBZ and BSA, suggesting recommendations for further studies and researches.

## Experimental

### Materials and chemicals

- Mebendazole (MBZ) was kindly provided by Alexandria Co. for Pharmaceuticals and Chemical Industries, Alexandria, Egypt
- Diazepam was kindly provided by Amoun Pharmaceutical Co., Cairo, Egypt.
- Indomethacin was received as a gift from Medical Union Pharmaceuticals Co, Ismailia, Egypt.
- Analytical grade Tris (hydroxymethyl) aminomethane hydrochloride (Tris–HCl) was bought from Sigma Aldrich (Germany).
- Bovine serum albumin (BSA) was also bought from Sigma Aldrich Co. (Chemie Gmbh, Munich, Germany), with batch No. SLBM6044V.

### Equipment and software

#### For Fluorescence measurements

Cary Eclipse Fluorescence Spectrophotometer was employes with Xenon flash lamp from Agilent Technologies (USA). A high voltage mode was used (900 V); the slit width was 5 nm and the smoothing factor was 20. Data were processed using prism.

When measuring the fluorescence spectrum, it is widely assumed that the inner filter effect (IFE) must be detected^12^. The following equation was used for correcting the fluorescence intensity with MBZ:

$$F_{cor} = F_{obs} \times e^{{\left( {A_{ex} + A_{em} } \right)/2}}$$

F_cor_: the corrected fluorescence intensity and, F_obs_ observed fluorescence intensity in the experiment. A^ex^ and A^em^ form the summation of MBZ absorbance at excitation 285 nm and the emission wavelength (λ), respectively.

A Hanna pH-meter (Romania) was used for adjusting pH.

#### UV–visible measurements

An operation of Shimadzu (Kyoto, Japan) UV-1601 PC using quartz cuvette, UV–visible double-beam spectrophotometer on a fast scan speed was conducted.

#### Infrared spectroscopy

FT-IR spectra are recorded using Thermo Fisher Scientific (168 Third Avenue Waltham, MA USA) on a Thermo Fisher Scientific Nicolet—iS10 FT-IR Spectrometer. It was equipped with a Ge/KBr beam splitter and a DTGS detector from 4000 to 1000 cm^−1^. For all measurements, 32 scans were recorded at 4 cm^−1^ resolution.

#### Molecular docking software

Downloading the crystal structure (3D) of BSA was made from the Protein Data Bank (www.rcsb.org), Code 4F5S^13,14^, and it was added into the Molecular Operating Environment software package (MOE 2019). This was conducted for pre-optimization by excluding water molecules and heteroatoms and adding the hydrogen atoms. ChemDraw Ultra 17.1 was used for obtaining the 3D structure of MBZ, and MOE 2019 software package in the compatible file format was used for acquiring the minimized energy structure and geometries of MBZ. A determination of binding pocket on BSA was detected. Keeping the receptor rigid, the triangle matcher principle was employed for the placement phase and the London dG function was employed for scoring using 30 runs for the drug.

## General procedure

### Preparation of stock solutions

BSA (2000 µM) stock solution was freshly prepared in bidistilled water. MBZ stock solution (100 µM) was prepared in formic acid. Tris hydrochloride buffer solution (pH = 7.4) was prepared in concentration of 20.0 mM in bidistilled water*.* All stock solutions were refrigerated at 4 °C and further diluted if necessary to get the working solutions.

### UV–visible absorption method

In this method, BSA concentration was kept constant at 40 μM, whereas the concentration of the drug was changed from 1 to 15 μM. At a temperature of 298 K, the UV region of 190–350 nm was scanned. The volume of solutions was completed using Tris buffer (20 mM, pH 7.4). To eliminate interference, the spectra were adjusted by subtracting the solvent absorption from both BSA and BSA-MBZ complex absorptions spectra.

### Fluorescence quenching method

BSA fluorescence quenching was studied at three temperatures: 298, 310, and 318 K along wavelength (300–500 nm) after excitation at 285 nm. BSA concentration was remained constant at 40 μM, while MBZ concentration was increased from 4 to 45 μM (4, 5, 10, 15, 20, 25, 30, 35, 40, 45 µM).

### Synchronous fluorescence method

BSA synchronous fluorescence spectra were scanned in the 200–320 nm range with different concentrations of MBZ (0–45 μM) after adjustment of Δλ at 60 nm for tryptophan and Δλ at 15 nm for tyrosine residues.

### FTIR method

Recording of FTIR spectra of BSA (500 μM) with and without MBZ was accomplished over the range of 1500–1800 cm^−1^ and setting BSA with the drug molar ratio at 1:1. Meanwhile, the value of absorbance of buffer and free MBZ solutions were measured and digitally subtracted from the spectra of the BSA-MBZ complex.

### Site probe studies

The competitive binding approaches were developed by using two approved site markers that are specific to the two main sites (Indomethacin for site I and diazepam for site II). The experiment was established by mixing equal concentrations of BSA and site marker, then setting them aside for 30 min to ensure maximal binding. Afterward, the mixture was titrated with different concentrations of MBZ, and the fluorescence emission spectra were recorded.

## Results and discussion

A detailed analysis of interactions between MBZ with BSA is not available in the literature. This tempted us to extensively characterize the interactions of MBZ with BSA using various spectroscopic approaches. The findings in this study indicate that MBZ binds with BSA at the site I. Thermodynamic investigations revealed that a stable ground state complex was formed between MBZ and BSA. The static mode of quenching was predominantly involved in the interaction of these two molecules. FTIR analysis confirmed that the binding of MBZ induced conformational changes in both the secondary and tertiary structure of BSA. Collectively, this paper characterizes the biochemical interactions occurring between BSA and MBZ and provides a comprehensive understanding of the pharmacodynamic properties of MBZ at the molecular level.

### UV–visible spectrophotometry

**Figure 2:**
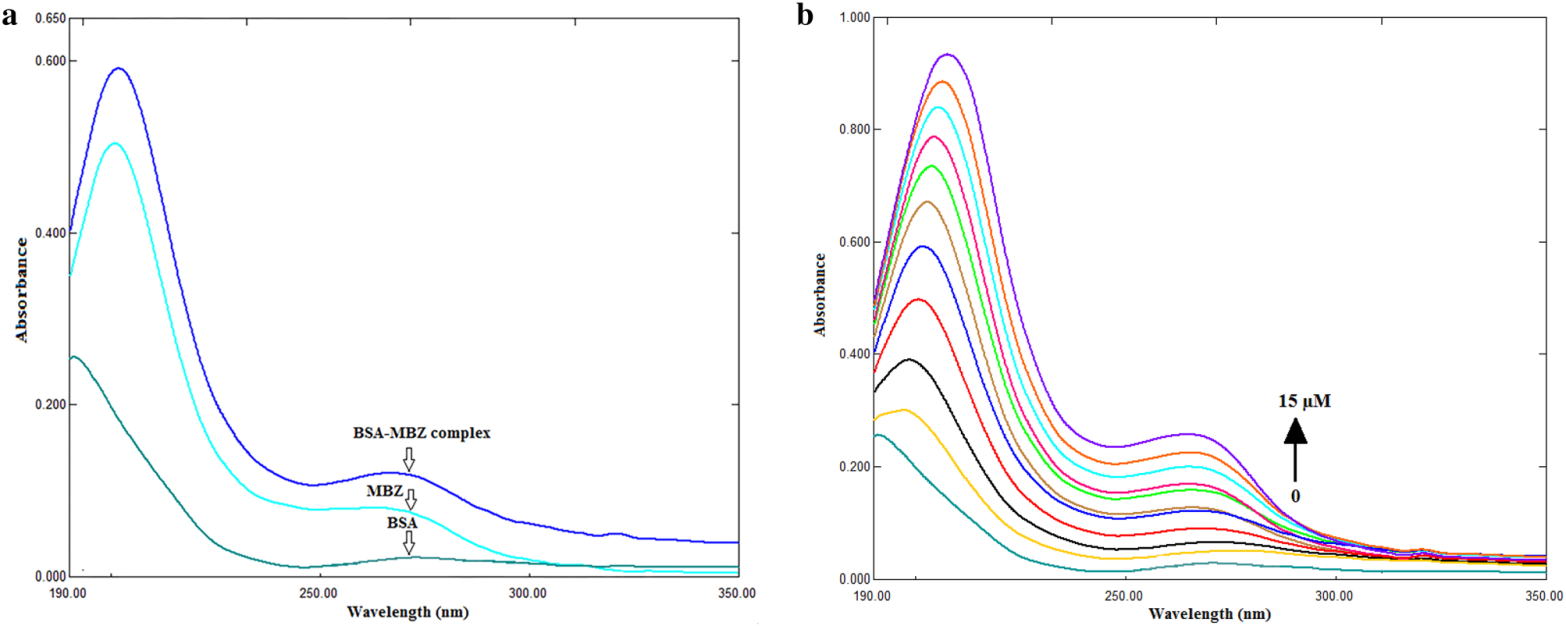
(**a**) UV–visible absorption spectra of MBZ (5 µM), BSA (40 µM), and BSA-MBZ complex at 298 K and pH 7.4. (**b**) UV–visible absorption spectra of BSA (C_BSA_ = 40 µM) and MBZ C_MBZ_ = (1, 2, 4, 5, 6, 8, 9, 10, 12, 15 µM) at 298 K and pH 7.4.

It is a reliable approach that may be used to investigate protein structural changes and the formation of protein-drug complexes^15,16^. The protein spectrum is susceptible to the microenvironment surrounding the chromophores^17^. Figures 2a,b show the influence of MBZ on the spectra of BSA. As illustrated in Fig. 2a, there is a detectable high absorption peak at 270 nm. While Fig. 2b reveals an increase in peak intensity with increasing MBZ concentrations confirming the interaction between MBZ and BSA.

### Fluorescence spectroscopy

**Figure 3:**
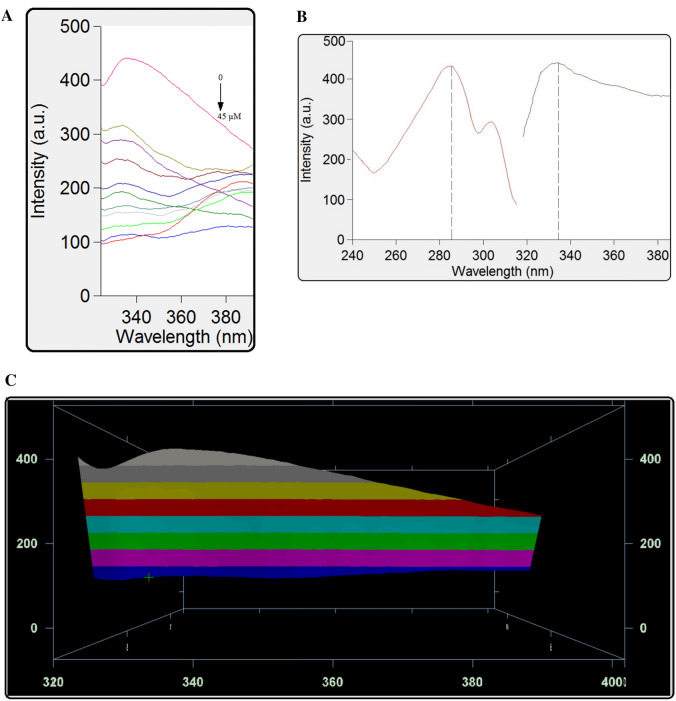
(**A**) Fluorescence spectra of BSA (40 µM) in presence of MBZ (4, 5, 10, 15, 20, 25, 30, 35, 40, 45 µM) at 298 K and pH 7.4. (**B**) Excitation (red curve) and emission (green curve) spectra for BSA 40 μM at 298 K and pH 7.4. (λ_em_) = 338 nm and (λ_ex_) = 285 nm. (**C**) 3D fluorescence spectra of BSA (40 µM) in presence of MBZ (4, 5, 10, 15, 20, 25, 30, 35, 40, 45 µM) at 298 K and pH 7.4.

Fluorescence spectroscopy is utilized to investigate drug-protein binding mechanisms to get information on the binding constant, number of binding sites, and thermodynamic parameters. Basically, changes in protein fluorescence intensity are detected at the maximum emission wavelength^12,18^. Adding MBZ to the BSA solution causes the formation of the MBZ-BSA, complex as shown in Fig. 3A–C. BSA fluorescence emission is quenched with increasing MBZ concentrations. It has two tryptophan residues and 18 tyrosine residues that display a strong bond upon protein excitation at 285 nm^19^.

Quenching of FI under these conditions is concentration-dependent and could be defined by the Stern–Volmer equation. The equation is adopted to evaluate the binding parameters at various temperatures:

1 $$\frac{{F_{o}{ }}}{F} = 1 + K_{sv}\left[ Q \right]$$

F_o_ and F: fluorescence intensities of BSA before and after binding with (MBZ). K_SV_ is the Stern–Volmer constant and [Q] is the concentration of the quencher.

**Figure 4:**
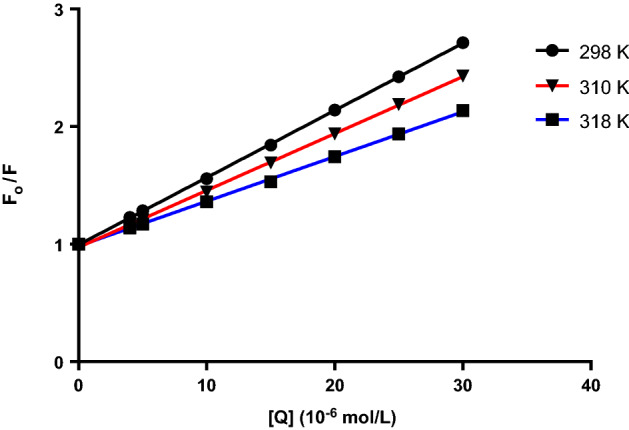
Stern–Volmer plots for BSA-MBZ system at various temperatures.

The plot of Eq. (1) in Fig. 4 was used to calculate the Stern–Volmer constant. Fluorescence quenching can be managed using one of three mechanisms: dynamic, static, and combination of the two mechanisms, but the principles of the two procedures vary since they are temperature-dependent^12,20^. The quenching constants in the dynamic quenching mechanism are predicted to increase along with rising temperatures, resulting in large diffusion coefficients. However, higher temperatures can cause a loss in complex stability, which can cause a decrease in the quenching constant value for static quenching^21^. Complex formation is proved by quenching rate constant (k_q_) values. The following equation is used for their calculation:

2 $${\text{}k_{q}} = \frac{{{\text{}K_{sv}}}}{{{\tau o}}}$$

where *k*_*q*_ is the bimolecular quenching rate constant, *τ*_*0*_* is* the average lifetime of the fluorophore in the excited state that is for a biomacromolecule 10^–8^ s^12,22,23^.

Figure 4 represents Stern–Volmer graphs at various temperature settings for MBZ quenching of BSA fluorescence, whereas Table 1 gives the K_SV_ and k_q_ values. The order of magnitude of the quenching rate constant k_q_ in this study was found to be 10^12^. Furthermore, the value of the bimolecular quenching rate constant was calculated and was found to be more than 2 × 10^10^ L mol^−1^ s^−1^^24^, suggesting a static quenching of the interaction between MBZ and BSA^25,26^.

**Table 1:** MBZ-BSA interaction parameters at various temperatures.

T (K)	K_sv_ (10^4^ L mol^−1^)	kq (10^12^ L mol^−1^ s^−1^)	R^2^	SD	%Error
298	5.657	5.657	0.9999	0.923	0.413
310	4.483	4.483	0.9994	1.232	0.551
318	3.623	3.623	0.9992	1.647	0.737

### Estimation of the binding constant and number of binding sites

**Figure 5:**
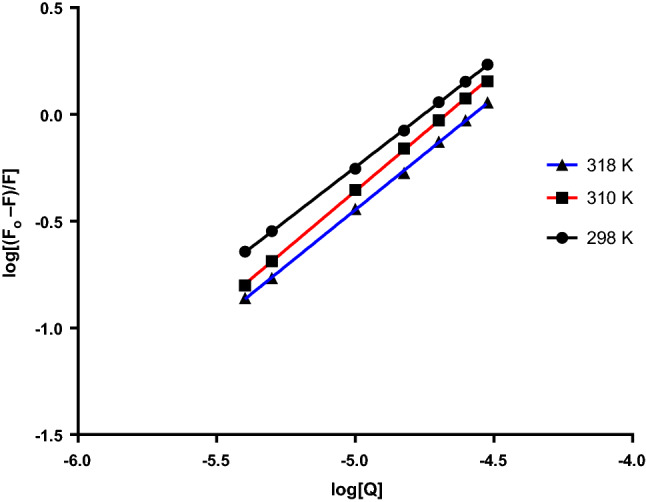
Plots of log (F_o_−F)/F versus Log [Q] at three temperature settings.

Both were determined using the so called Modified Stern–Volmer equaton^27^:

3 $$\log \frac{F_{o} - F}{F} = \log K_{b} + n \log \left[ Q \right]$$

where K_b_ and n are the binding constant and number of the binding site, respectively. Both can be obtained by plotting log $\left( {\frac{F_{o} - F}{F}} \right)$ against log [Q]. The values of log *K*_*b*_ and n are provided by the intercept and slope of Fig. 5, respectively. Table 2 shows the results attained at various temperature settings. The binding ratio of BSA to MBZ was found to be nearly 1:1, and the binding constant decreases upon increasing temperature.

**Table 2:** MBZ-BSA complex binding characteristics at different temperatures.

T (K)	K (L mol^−1^)	n	R^2^
298	5.67 × 10^4^	1.003	0.9999
310	4.48 × 10^4^	1.091	0.9997
318	3.76 × 10^4^	1.049	0.9995

### Determination of thermodynamic parameters

The drug-protein binding involves four main forces, viz: hydrophobic forces, hydrogen bonding, electrostatic interactions, and van der Waals forces. The thermodynamic parameters, including enthalpy change (ΔH^ο^) and entropy change (ΔS^ο^) describe the binding forces in the process^28^. Ross and Subramanian investigated the relationship between changes in thermodynamic parameter values and binding forces^29^. When ΔH^ο^ > 0 and ΔS^ο^ > 0, hydrophobic forces dominate, while van der Waals forces and hydrogen bonding dominate the process when ΔH^ο^ < 0 and ΔS^ο^ < 0. Electrostatic forces are considered the main forces when ΔH^ο^ < 0 and ΔS^ο^ > 0. In case of slight temperature differences, Van’t Hoff equation can be utilized for determining ΔH^ο^ and ΔS^ο^^29,30^.

4 $$\ln K = - \frac{{\Delta {\text{H}}^{^\circ } { }}}{{{\text{RT}}}} + \frac{{\Delta {\text{S}}^{^\circ } }}{{\text{R}}}$$

where R is the gas constant and *K*_b_ is the binding constant at temperature T.

**Figure 6:**
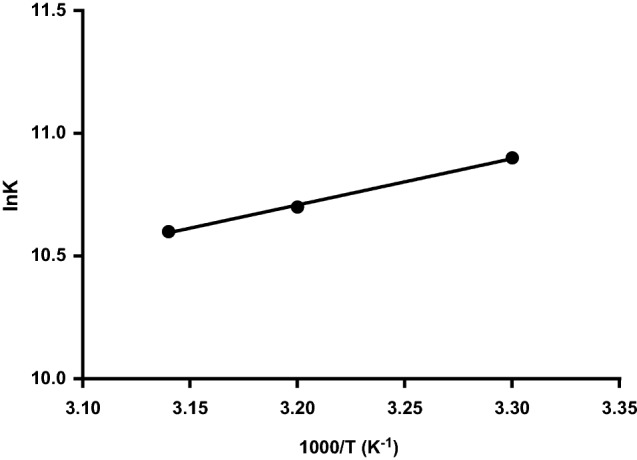
Van’t Hoff plot for BSA- MBZ binding.

A straight line is obtained by plotting ln *K*_*b*_ versus 1/ *T*. The resulting slope represents the enthalpy change (ΔH^ο^), whereas the intercept represents the entropy change (ΔS^ο^) (Fig. 6). The following equation can be used to calculate free energy change (ΔG^ο^):

5 $$\Delta G^{^\circ } = \Delta H^{^\circ } - T\Delta S^{^\circ }$$

As presented in Table 3, the negative free energy change (ΔG^ο^) and the positive entropy change (ΔS^ο^) reveal the spontaneous binding of MBZ-BSA complex. Furthermore, the main force in this reaction is the hydrophobic interaction. An endothermic reaction is revealed by associating the positive value of enthalpy change (ΔH^ο^) and the increasing K values with the three different temperatures.

**Table 3:** Thermodynamic parameters of MBZ-BSA Interaction at pH 7.4.

T (K)	Δ*H*° (kJ/mol)	Δ*G*° (kJ/mol)	Δ*S*° (J mol^−1^ K^−1^)	R^2^
298	158.51	− 31.83	638.75	0.9978
310		− 39.49		
318		− 44.61		

### Synchronous fluorescence spectra of BSA

**Figure 7:**
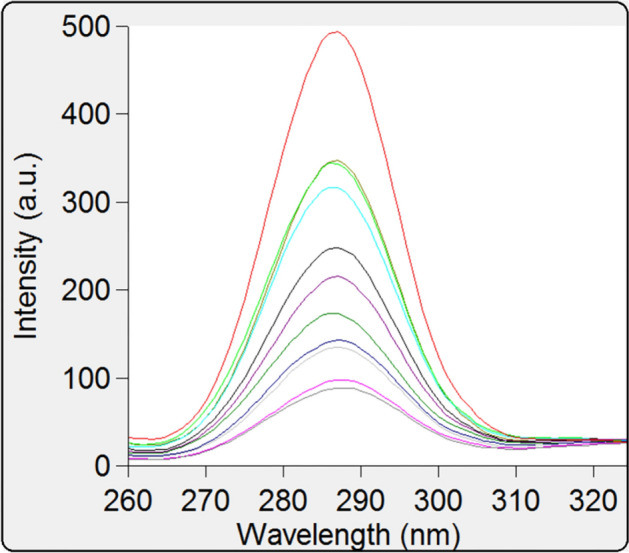
Synchronous fluorescence spectra of BSA (40 µM) at Δλ 15 nm in existence of MBZ (4, 5, 10, 15, 20, 25, 30, 35, 40, 45 µM).

**Figure 8:**
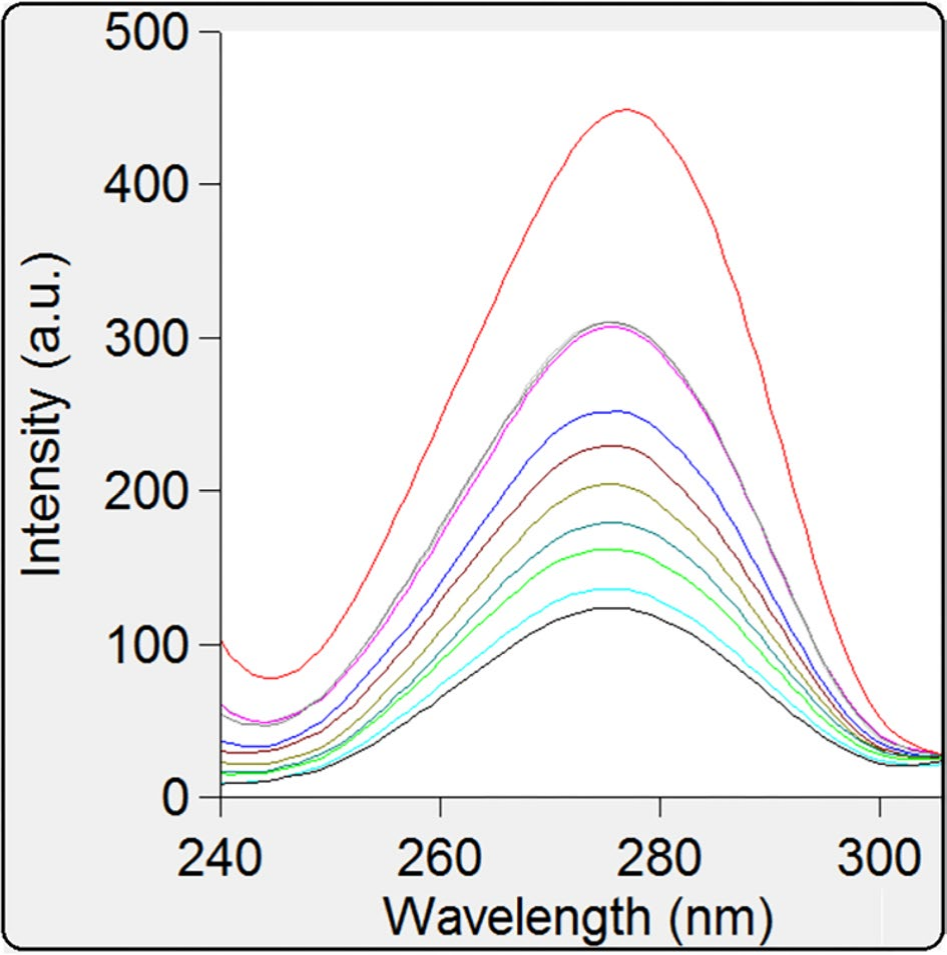
Synchronous fluorescence spectra of BSA (40 µM) at Δλ 60 nm in existence of MBZ (4, 5, 10, 15, 20, 25, 30, 35, 40, 45 µM).

The synchronous spectrofluorimetric approach has many benefits: spectral simplicity, sensitivity, spectral bandwidth, as well as minimizing and preventing various disturbing effects. It is highly beneficial to accomplish the emission wavelength shift in order to investigate the protein's microenvironment^31^. In BSA, Trp and Tyr residues are revealed by the use of synchronous fluorescence spectra at wavelength intervals (Δλ) of 60 nm and 15 nm, respectively. It is further displayed in the synchronous fluorescence spectra of BSA at Δλ = 15 nm (Fig. 7) and at Δλ = 60 nm (Fig. 8) with different MBZ concentrations. In Fig. 7, at Δλ 15 nm, it was revealed that the maximum emission range for the studied concentrations remained unchanged, while at Δλ 60 nm, a minor blue shift (277–274 nm) was noticed as illustrated in Fig. 8. The polarity surrounding tryptophan residues was observed to be attenuated, suggesting that it occurred in a hydrophobic environment. Therefore, the conformation of BSA after binding with MBZ is suggested by the observed spectral change^32^.

### Site probe studies

**Figure 9:**
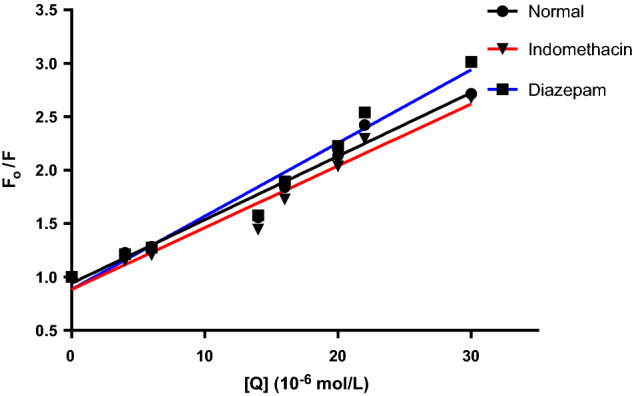
Stern–Volmer plots for BSA quenching by MBZ before and after adding site markers.

BSA is composed of 3 domains: I, II, and III, with each domain, further subdivided into two subdomains A and B^3,33^. An investigation of site marker measurements was conducted to determine the MBZ binding site in BSA. According to Sudlow et al.^34^, drugs bind to albumin at sites I or II, which are situated inside the hydrophobic regions of subdomains IIA and IIIA, respectively. Indomethacin binds to site I and diazepam binds to site II^35,36^. For calculation of the binding parameters resulting from the impact of both site markers on MBZ-BSA interaction, the Stern–Volmer equation was applied and the plot is shown in Fig. 9. The binding constants of the interaction between MBZ and BSA were calculated in the presence of the two site markers as shown in Table 4. Noticeably, MBZ binding with BSA is weakened in the presence of indomethacin. However, the binding constant remained unchanged upon adding diazepam. Hence, it may be deduced that MBZ-BSA binding occurs at the site I of subdomain IIA. There is an agreement between these outcomes and the results of the molecular docking technique presented in “Molecular Docking”section.

**Table 4:** Stern volmer quenching constants and bimolecular quenching rate constants for MBZ- BSA interaction in presence of site markers.

Site marker	K_sv_ (10^4^ L mol^−1^)	kq (10^12^ L mol^−1^ s^−1^)	SD	%Error
BSA + MBZ	5.657	5.657	0.923	0.413
BSA + MBZ + IND	4.743	4.743	1.017	0.455
BSA + MBZ + DIA	5.94	5.94	0.908	0.406

### FT-IR spectroscopy

**Figure 10:**
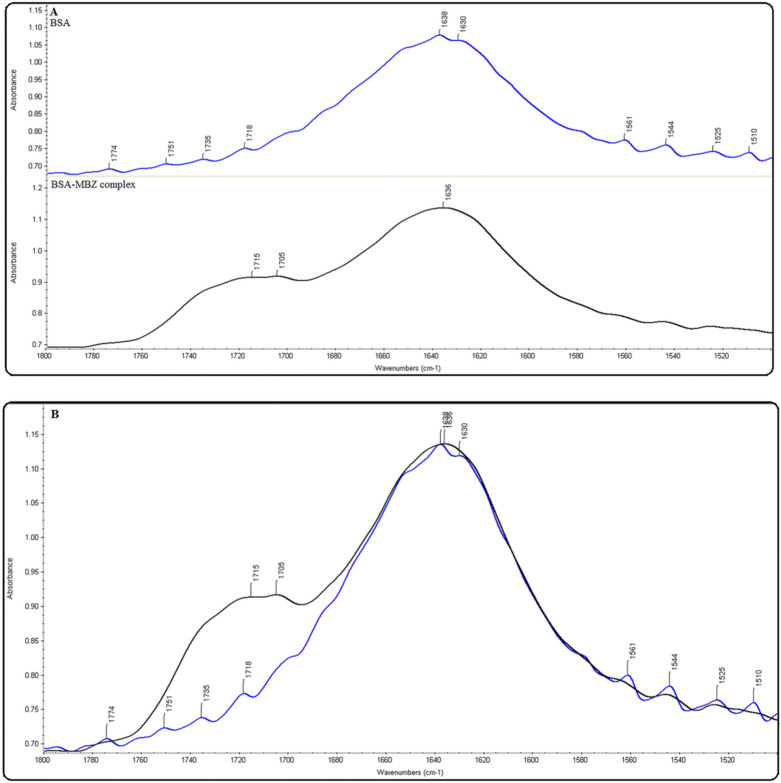
Free BSA and BSA-MBZ complex FTIR spectra at 298 K at pH 7.4.

FT-IR spectroscopy is effective for studying protein dynamics and secondary structures^37^. Confirmation of the interaction between MBZ and BSA is revealed by FT-IR spectra in Fig. 10. Amide bands are the main constituent of the protein’s infrared spectra, as they cause many peptide moiety vibrations. The amides I and II peaks, which occur in the region of 1600–1700 cm^−1^ and 1500–1600 cm^−1^, respectively, are often employed in secondary protein structure studies. The amide I band is more susceptible to alterations than the amide II band, which makes it useful for examining the protein's secondary structure. The amide I peak location was shifted from 1638 to 1636 cm^−1^ after the MBZ reaction, suggesting that the secondary protein structure alters during MBZ-BSA interaction^38^.

### Fluorescence resonance energy transfer (FRET) between MBZ and BSA

**Figure 11:**
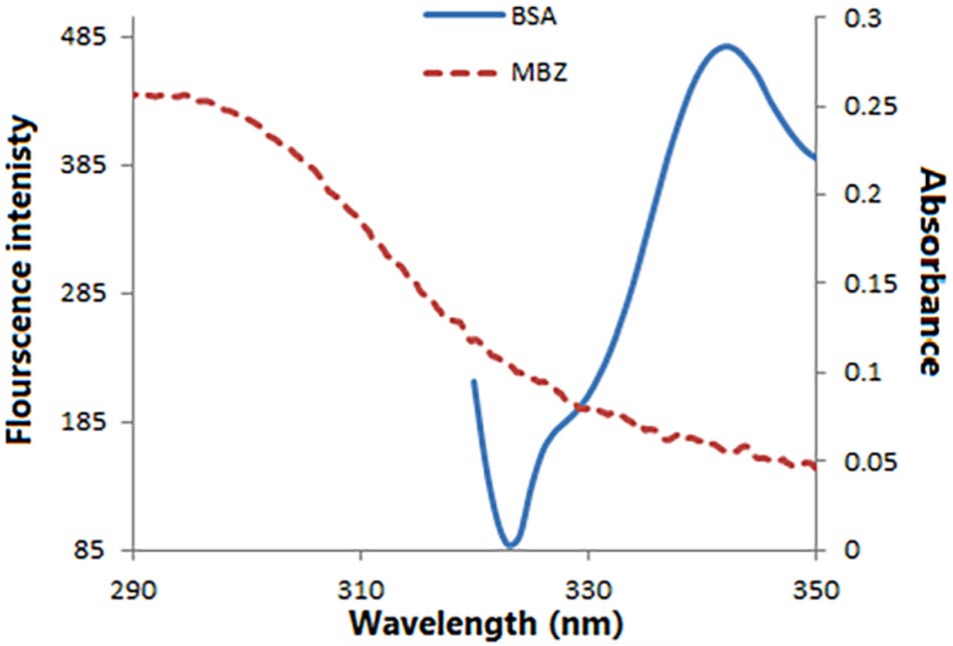
Spectral overlap between the BSA fluorescence emission spectrum (40 µM) and the absorption spectrum of MBZ (15 µM) at 298 K and pH 7.4.

FRET is a phenomenon that involves transferring non-radiative energy along with the distance between a donor molecule (BSA) and an acceptor molecule (MBZ). Resonance takes place in a distant-dependent manner through dipole–dipole coupling between the two fluorophores, in absence of molecular collision or thermal energy conversion. The non-radiative energy transfer theory proposed by Fӧrster states that many factors have an influence on FRET^39^:

1. As illustrated in Fig. 11, there is an overlapping between the donor's emission spectra and the acceptor's absorption spectrum.
2. The magnitude of this overlap is represented by the spectral overlap integral (*J*).
3. Both the acceptor and donor molecules should have parallel transition dipole orientations.

To determine the efficiency of energy transfer (*E*), the following formula was applied:

6 $$E = 1 - \frac{F}{{F_{o} }} = \frac{{R_{o}^{6} }}{{R_{0}^{6} + r^{6} }}$$

where *F*_*o*_ and *F* represent BSA fluorescence intensity in absence and presence of MBZ, respectively. *r* signifies the distance between the BSA and the MBZ. *R*_*o*_ is the Fӧrster distance where the energy transfer is 50% efficient.

Calculation of the value of *R*_*o*_ was obtained from Eq. (7):

7 $$R_{o}^{6} = 8.8 \times 10^{ - 25} k^{2} N^{ - 4} \Phi {\text{J}}$$

where *k*^*2*^ is the dipole angular orientation of each molecule, *N* is the refractive index of the medium, Ф is the fluorescence quantum yield of the donor and *J* is the spectral overlap integral of the fluorescence emission spectrum of the donor and the absorption spectrum of the acceptor and was assessed by applying in the Eq. (8):

8 $$J = \frac{{\smallint F\left( \lambda \right)\varepsilon \left( \lambda \right)\lambda^{4} \Delta \lambda }}{\smallint F\left( \lambda \right)\Delta \lambda }$$

where *F* (λ) is the fluorescence intensity of the fluorescent donor of wavelength λ. (λ) is the molar absorption coefficient of the acceptor at wavelength λ.

In this study, *k*^*2*^ = 2/3. *N* = 1.336 and *Ф* = 0.15. The spectral overlap integral (*J*) is determined over the range of 300–450 nm. The values of *J* = 8.69 × 10^–16^ cm^3^ L mol^−1^^40^, *E* = 0.42, *R*_*o*_ = 1.70 nm and *r* = 1.79 nm can be computed from Eqs. (6) – (8). The average distance between BSA and MBZ is less than 8 nm suggesting that the energy transfer occurs^41^.

### Molecular docking

**Figure 12:**
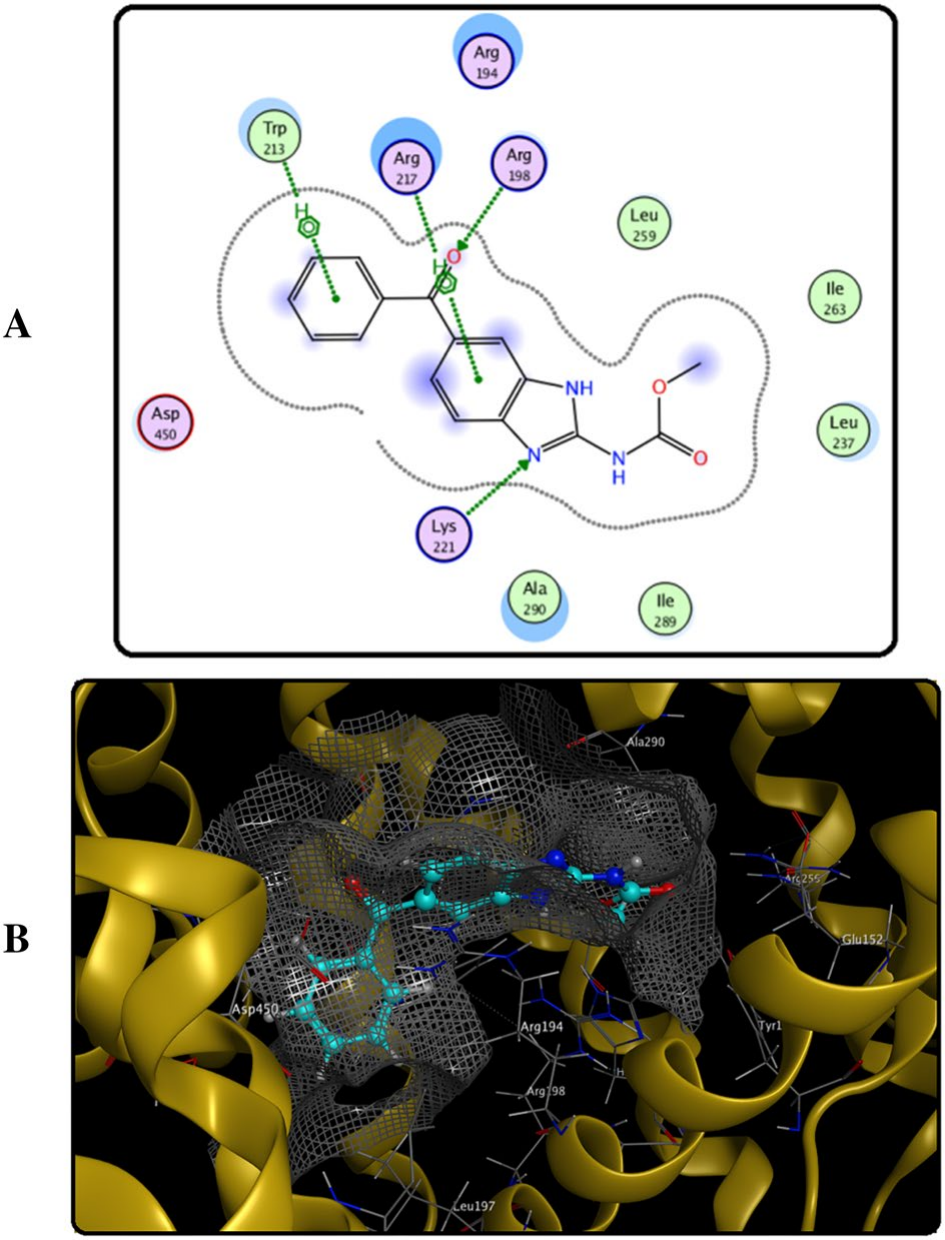
(**A**) Diagram for the amino acid residues incorporated in MBZ-BSA interaction within the binding pocket of BSA site 1, (**B**) 3D structure of the binding interaction between MBZ-BSA.

A useful simulation tool used in evaluating the nature of the drug-protein binding is Molecular docking. It was used to validate our data obtained about MBZ binding affinity and interactions at its binding site in BSA. Results are obtained from this technique, confirming the previously reported studies of the site marker displacement, i.e., the binding of MBZ with BSA at the site I of subdomain IIA (Fig. 12A,B). The configuration of MBZ was recorded after binding with BSA, revealing binding energy of − 6.9 kcal mol^−1^. This MBZ conformer is located within the active site residues Arg194, Arg198, Ala290, Trp213, Arg217, Leu259, Leu237, lys221, Ile289, Ile263 and Asp450 (Fig. 12A,B). Furthermore, it was shown that MBZ formed hydrophobic interaction with BSA through residue Trp213. Results from molecular modeling correlate with the previous experimental results proving the occurrence of MBZ-BSA binding through hydrophobic interaction.

## Conclusion

In this proposed study, an evaluation of the MBZ-BSA binding interaction is established under physiological conditions with the use of multi-spectroscopic and molecular docking approaches. Intensive research has been conducted using a variety of techniques. MBZ-BSA binding was found to be static quenching. The thermodynamic parameters for MBZ-BSA interaction were also determined. The binding of MBZ-BSA was a spontaneous endothermic process, with hydrophobic interaction being the dominating factor in the process. In accordance with the site marker technique which was confirmed by molecular docking studies, MBZ-BSA binding occurs at site I in subdomain IIA. This study provides valuable insights into a better understanding of the pharmacokinetic profile of MBZ. The data presented in this work might help in understanding the molecular mechanisms underlying the harmful side effects of MBZ, thereby improving its pharmacological and clinical efficacy. The results proved the binding relationship between MBZ and BSA as well as the ease of transportation and elimination, suggesting recommendations for further studies and research. This study can provide experimental evidence of MBZ toxicity and supply basic data for its safe use.

## Data Availability

All data generated or analyzed during this study are included in this published article.
